# Falling Between the Cracks: Experiences of Black Dementia Caregivers Navigating U.S. Health Systems

**DOI:** 10.1093/geroni/igab046.3490

**Published:** 2021-12-17

**Authors:** Karah Alexander, Sloan Oliver, Fayron Epps

**Affiliations:** 1 Nell Hodgson Woodruff School of Nursing at Emory University, Atlanta, Georgia, United States; 2 Emory University, Atlanta, Georgia, United States

## Abstract

In addition to numerous care responsibilities, family caregivers are expected to navigate health systems and engage in healthcare management tasks on behalf of their persons living with dementia (PLWD). These challenging tasks pose additional difficulties for Black dementia caregivers. Due to the centuries-old, disadvantaged social history of Black Americans, several unique stressors, vulnerabilities, and resources have emerged which inform and affect Black dementia caregivers’ experiences and well-being. Focus groups were held with Black caregivers (N = 19) from the United States (U.S.) to explore the unique experiences and perspectives of this population navigating the U.S. health system on behalf of their PLWD. Five overarching themes were developed during thematic analysis: Forced Advocacy, Poor Provider Interaction, Payor Source Dictates Care, Discrimination, and Broken Health System. Black dementia caregivers unanimously concurred that the health system that they experience in America is “broken.” Gaps in the health system can lead to people [as one caregiver passionately expressed] “falling between the cracks,” in terms of care, services, and resources needed. Caregivers agreed that class, sex, utilizing public health insurance, and being a “person of color” contribute to their difficulties navigating the health system. Caregivers perceived being dismissed by providers, forcing them to advocate for both themselves and their PLWD. Healthcare providers and researchers can utilize these findings to improve the experiences and healthcare outcomes of Black patients with dementia and their caregivers. Additionally, these findings can lead to the development of culturally tailored caregiver education programs.

